# Can Microneedle-Nanocarrier Platforms Deliver True Tolerogenic Immunotherapy for Psoriasis?

**DOI:** 10.3390/vaccines14090784

**Published:** 2026-09-08

**Authors:** Mingpeng Feng, Hong Zhou

**Affiliations:** 1 Medicine & Technology College, Zunyi Medical University, Zunyi 563000, China; destinyx2023@outlook.com; 2 USDA/ARS Children’s Nutrition Research Center, Department of Pediatrics, Baylor College of Medicine, Houston, TX 77030, USA

**Keywords:** psoriasis, tolerogenic vaccines, transdermal nanovaccines, microneedles, transdermal delivery, cutaneous immunomodulation, regulatory T cells, IL-23/IL-17 axis

## Abstract

Psoriasis is a chronic, relapsing skin disease driven by failed immune tolerance toward self-antigens and sustained IL-23/IL-17 signaling. Systemic drugs and biologics control disease in many patients, yet they do not restore lasting tolerance, and most patients relapse after treatment withdrawal. Tolerogenic vaccines aim to re-educate the cutaneous immune system: when administered under non-inflammatory conditions, they push antigen-presenting cells toward tolerogenic dendritic cells (tDCs) and regulatory T cells (Tregs) instead of pathogenic effectors. Because the hyperkeratotic stratum corneum of psoriatic plaques blocks macromolecular vaccines, microneedle (MN) arrays can deposit nanocarriers directly into the dermal-epidermal junction. The narrative review asks whether MN-delivered tolerogenic nanovaccines can advance psoriasis therapy beyond broad immunosuppression. We first define tolerogenic vaccination and explicitly distinguish antigen-specific tolerogenic vaccines, antigen-independent immune-reprogramming nanocarriers, and conventional local drug delivery, then survey MN engineering and nanocarrier architectures, the immunomodulatory pathways involved, and translational barriers spanning manufacturing, safety, and regulation. Because most evidence is preclinical, the level of evidence is reported for each platform, and the unresolved autoantigen problem, the limitations of imiquimod-based models, and the need for human-relevant validation are emphasized.

## 1. Introduction

Roughly 2–3% of people worldwide live with psoriasis, and the disease exacts a heavy physical, psychological, and economic toll [1,2,3,4]. Its pathogenesis arises from an interplay of genetic susceptibility, epigenetic regulation, environmental triggers, and disturbed cutaneous immune networks [5,6,7,8]. The cutaneous microbiome has emerged as a key modulator of skin immune homeostasis, with dysbiosis increasingly recognized as a contributor to inflammatory skin diseases [9]. In genetically prone individuals, epidermal injury or stress can set off a cascade in which keratinocytes and infiltrating immune cells release antimicrobial peptides (AMPs) and self-nucleic acids. These mediators are increasingly seen as disease amplifiers, and AMPs shuttled by extracellular vesicles may carry inflammatory signals across the skin [10,11,12]. Dendritic cells, the principal antigen-presenting cells of the skin, then guide T helper responses along a pathogenic trajectory. Most investigators now consider the IL-23/IL-17 axis the dominant effector pathway in plaque psoriasis. IL-17-secreting T cells, in particular Th17 cells and dermal gamma-delta T cells, drive keratinocyte overgrowth, chemokine release, and sustained neutrophilic inflammation, closing a self-amplifying DC-T cell-keratinocyte loop [2,10,13,14]. The clinical relevance of this pathway is supported by the success of IL-17/IL-17RA- and TYK2-targeted drugs, whether given systemically or in emerging topical formulations [10,15,16]. From a vaccinological standpoint, psoriasis reflects a breakdown of cutaneous immune tolerance to self-antigens rather than mere excess of effector inflammation [10,17,18].

Throughout this review, the term tolerogenic vaccine refers to a therapeutic, tolerance-inducing strategy, not to prophylactic immunization against infectious pathogens [12,19]. To make this distinction operational and apply it consistently throughout the text, tables, and figures, we classify formulations into three categories: (i) antigen-specific tolerogenic vaccines, which co-deliver a defined self-antigen or its epitopes together with tolerogenic signals and aim to elicit antigen-specific Tregs or deletional tolerance; (ii) antigen-independent immune-reprogramming platforms, which deliver immunomodulatory cargoes, including small molecules, nucleic acid therapeutics, or cytokines, to remodel the cutaneous microenvironment toward tolerance even without a defined autoantigen; and (iii) conventional local drug delivery, which suppresses inflammation or keratinocyte proliferation without engaging antigen-specific tolerance mechanisms. A formulation is classified as a tolerogenic vaccine only if it is designed to induce durable, preferably antigen-specific immune tolerance, supported by endpoints such as Treg induction, T-cell anergy or deletion, or protection against relapse after treatment withdrawal; transient anti-inflammatory efficacy alone is not sufficient. Mechanistically distinct as the first two categories are, they converge on restoring cutaneous immune homeostasis, and we keep them apart from category (iii) because their regulatory pathways, manufacturing demands, and clinical development trajectories differ [12,19,20]. The aim in psoriasis is to silence autoreactive T cell clones and rebuild immune homeostasis without the broad immunosuppression produced by systemic agents [21,22,23]. Existing systemic and biologic therapies still suffer from adverse effects, resistance, high cost, and inconsistent responses, and this is what drives the search for tolerance-restoring alternatives [24,25]. The clinical motivation is easy to state. After treatment withdrawal, relapse rates as high as approximately 90% have been reported in some analyses, a figure commonly derived from biologic withdrawal studies that varies considerably across drug classes, patient populations, and follow-up durations, although the exact figure depends on the study population and treatment modality [26]; the incomplete durability of response is widely attributed to pathogenic CD8^+^ tissue-resident memory T (TRM) cells that persist in clinically clear skin, where standard therapies cannot reach them [14,26]. The prophylactic immunization runs through the entire review and matches how tolerogenic vaccines are understood in autoimmunology [19].

**Figure 1:**
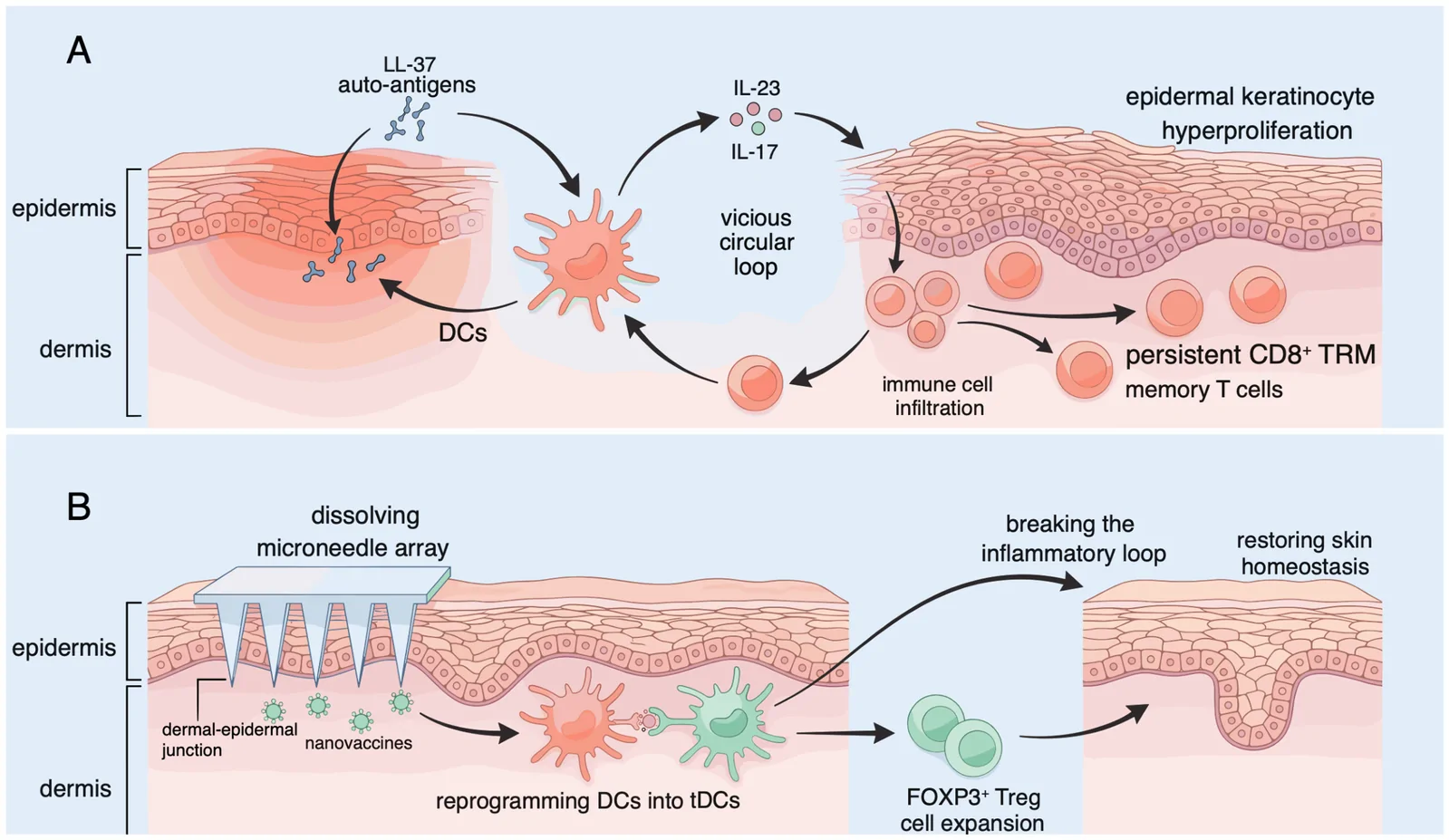
Immunopathological cascade of psoriasis and therapeutic resolution via tolerogenic nanovaccines. (**A**) The pathological vicious cycle in psoriatic skin. LL-37 autoantigens activate dendritic cells (DCs), initiating the IL-23/IL-17 signaling axis. Effector T cells secrete IL-17A, driving epidermal keratinocyte hyperproliferation and recruiting infiltrating immune cells. This inflammatory environment sustains persistent CD8^+^ tissue-resident memory T (TRM) cells, forming a self-amplifying circular loop that maintains chronic inflammation. (**B**) Therapeutic intervention using a dissolving microneedle array. The microneedles breach the stratum corneum to deliver nanovaccines directly into the dermal-epidermal junction. These nanovaccines reprogram mature DCs into tolerogenic phenotypes (tDCs), which subsequently promote the peripheral expansion of FOXP3+ regulatory T cells (Tregs). This expanded Treg population breaks the inflammatory loop and restores long-term cutaneous immune homeostasis.

The skin already carries the machinery needed for tolerance induction. Epidermal Langerhans cells, dermal DCs, macrophages, and resident memory T cells all reside there [21,22,27]. Topical vaccination for psoriasis nonetheless runs into two obstacles. The first is physical. Hyperkeratosis and parakeratosis thicken the stratum corneum, and its lipid-rich brick-and-mortar architecture impedes hydrophilic macromolecules, proteins, and nucleic acids [14,20,28,29]. The second is biochemical. Reactive oxygen species (ROS), matrix metalloproteinases, and proteases saturate the inflamed tissue and can destroy unprotected biological payloads before they ever reach the viable epidermis [30,31,32,33]. Together these barriers explain why creams and ointments achieve poor penetration into plaque skin, a problem shared by small-molecule and biologic therapies [34,35,36]. Microneedle (MN) arrays were designed to circumvent the stratum corneum while retaining the convenience of topical application [37,38,39]. Their micrometer-scale needles, roughly 200 to 1000 μm tall, create transient microchannels that deliver vaccines and nanomedicines into the APC-rich dermal-epidermal junction (DEJ) with little pain and no biohazardous sharps waste [39,40,41]. Wrapping active agents in nanocarriers adds another layer of benefit, shielding them from enzymatic degradation, prolonging local residence, and permitting receptor-mediated targeting of cutaneous APCs [42,43,44]. The MN-plus-nanovaccine concept therefore joins biomaterials engineering to translational immunology and redirects psoriasis care from systemic immunosuppression toward localized, antigen-specific immune re-education [36,45,46]. The immunopathological cascade of psoriasis and the therapeutic resolution via tolerogenic nanovaccines are illustrated in Figure 1.

The review asks whether microneedle-delivered tolerogenic nanovaccines can carry psoriasis therapy beyond broad immunosuppression toward antigen-specific immune re-education, and which mechanistic, engineering, and translational constraints stand in the way of clinical use [3,7,45], and all Flowchart of the literature screening and filtering workflow derived from Pubmed is included in Supplementary File and visulized in Figure S1.

## 2. Engineering Design and Classification of Microneedle Vaccine Platforms

**Figure 2:**
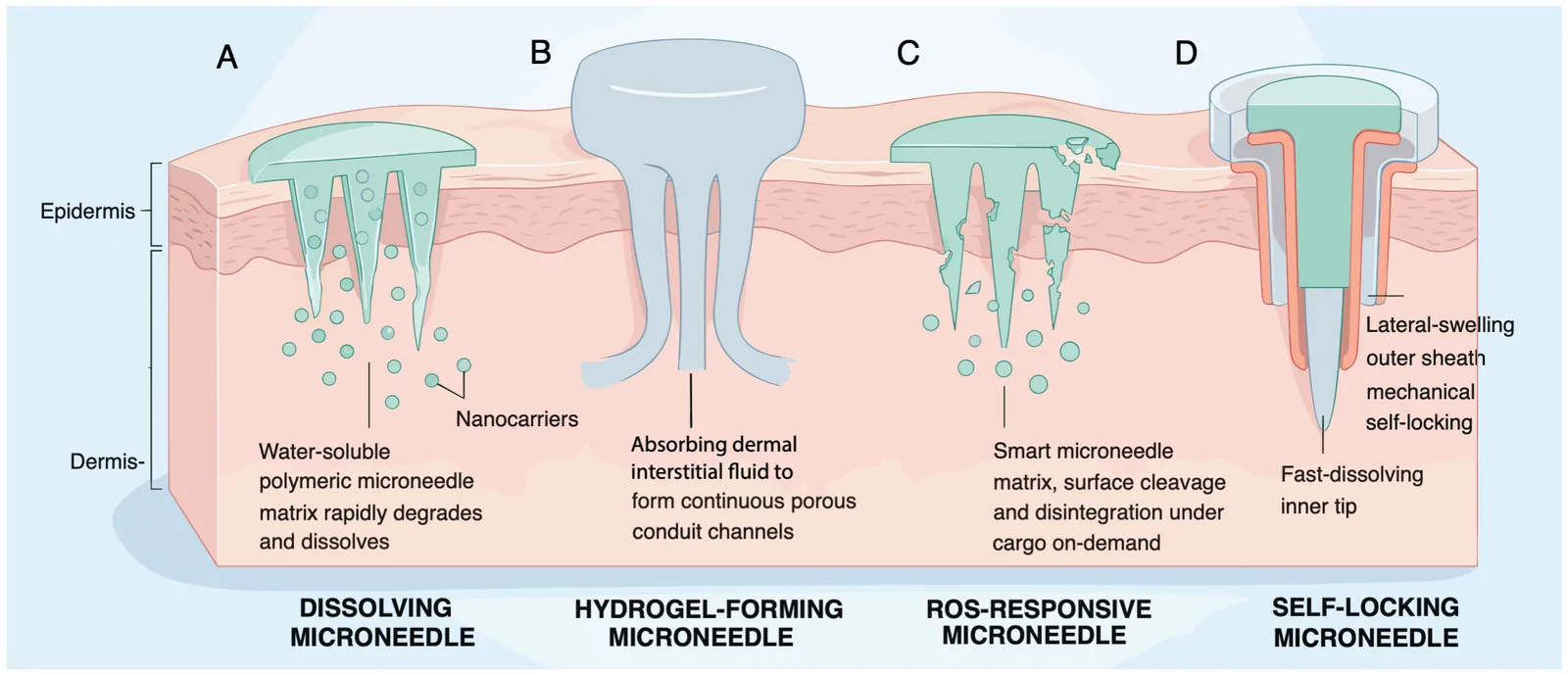
Engineering mechanics and payload release profiles of transdermal microneedle platforms. Cross-sectional schematic illustrating microneedle arrays breaching hyperkeratotic skin layers to deliver nanovaccines into the dermal-epidermal junction. (**A**) Dissolving microneedles (DMNs) constructed from soluble polymers rapidly degrade within the dermis to release cargo. (**B**) Hydrogel-forming microneedles (HFMNs) swell upon absorbing interstitial fluid, creating continuous fluid conduits for sustained diffusion from a backing reservoir. (**C**) Stimuli-responsive smart microneedles undergo matrix cleavage under reactive oxygen species (ROS) oxidation for on-demand payload release. (**D**) Self-locking bilayer microneedles anchor mechanically within the epidermis via lateral swelling while delivering therapeutic agents into the dermis.

Penetration depth and drug-release behavior both hinge on the mechanical properties, material composition, and degradation kinetics of the microneedle itself [34,38,47]. From a vaccinology perspective, these platforms convert the psoriatic plaque into an immunization site. Transient microchannels opened by the MN array let the tolerogenic cargo, together with nanocarrier-derived signals that condition resident antigen-presenting cells, reach the skin [17,37,38,39]. Several platform families have now emerged, distinguished by their insertion mechanics and payload release profile (Figure 2).

### 2.1. Dissolving and Hydrogel-Forming Microneedles

Dissolving microneedles (DMNs) are fabricated from water-soluble, biocompatible polymers, with hyaluronic acid (HA), carboxymethyl cellulose, polyvinylpyrrolidone, and polyvinyl alcohol being the most commonly used. After insertion the needles imbibe interstitial fluid, dissolve over minutes to hours, and unload their cargo into the epidermis and superficial dermis [40,48,49]. Preclinical psoriasis studies have used DMNs to deliver methotrexate (MTX), tacrolimus (TAC) nanocrystals, and a range of immunomodulatory nanoparticles, with drug retention in skin exceeding that of standard ointments [49,50,51]. Tip-loaded dissolving arrays have also carried macromolecular biologics, including TNF-alpha antibodies [52], and related patches show anti-inflammatory activity in other inflammatory skin conditions, with cyanocobalamin [53], and cyclosporin A [54]. Low-molecular-weight HA is prized as a structural matrix because it engages CD44, a receptor overexpressed by psoriatic keratinocytes and inflammatory cells, so the patch gains a targeting function alongside its structural one [55,56,57]. Supramolecular DMN designs accommodate hydrophobic glucocorticoids that dissolve poorly in ordinary water-soluble matrices [58], while trilayer dissolving patches sustain the release of poorly soluble actives, calcipotriol nanosuspensions among them [4]. Hydrogel-forming microneedles (HFMNs) rely on crosslinked hydrophilic polymer networks that swell after insertion and establish continuous hydrogel conduits between a drug reservoir and the dermis [59]. Because the swollen patch is withdrawn intact, no polymer residue remains, and HFMNs tolerate higher doses or extended-release schedules than single-use DMNs [41,59]. Their principal drawbacks are the backing reservoir and a lag phase before steady-state diffusion sets in [59]. For psoriasis specifically, HFMN concepts trail DMNs, although investigators are actively testing them for painless intradermal delivery of phytochemicals and macromolecular payloads [37,41].

### 2.2. Stimuli-Responsive, Bilayer, and Self-Locking Microneedles

Elevated ROS, hypoxia, and protease activity in lesional skin make microenvironment-responsive MNs appealing, because they can release cargo on demand, scaled to disease severity [31,60,61]. ROS-responsive gel-based MN patches carrying MTX and epigallocatechin gallate keep drug levels high for longer and adapt to relapsing disease in animal studies [31]. ROS-labile bilayer systems offer another route. A PVA-TSPBA tip loaded with MTX sits above a gelatin methacryloyl (GelMA) backing that slowly emits cerium oxide (CeO_2_) nanozymes, giving spatiotemporal control over anti-inflammatory and antioxidant activity [60]. ROS-responsive prodrug nanoassemblies and ROS-sensitive hydrogels loaded with natural products, including Momordin Ic, have likewise been folded into dissolvable MN patches for site-specific release [32,61,62]. Bilayer and self-locking architectures solve a second problem: patch detachment from flaking, hyperkeratotic plaques. Wang et al. created a self-locking MN patch with a rapidly dissolving polyvinyl alcohol-based inner compartment that carries the TYK2 inhibitor deucravacitinib and dampens the IL-23/IL-17 pathway, plus an outer compartment delivering calcipotriol. Hydration makes the outer matrix swell and anchor the needles inside the lesion for sustained antiproliferative action [63]. Related bilayer patches pairing MTX with dexamethasone, or methylprednisolone with upadacitinib-loaded mesoporous silica nanoparticles, split rapid anti-inflammatory and sustained antiproliferative functions along similar lines [64,65]. Core–shell MN designs with in situ calcium ion-coordinated self-assembly have been proposed to balance mechanical strength against controllable release in thickened skin [66].

### 2.3. Micro-Engine-Driven and Other Advanced Microneedle Platforms

Passive diffusion can take MN-delivered payloads only so far into thick plaques. Zheng et al. went a step further with a pneumatic MN patch driven by live Enterobacter aerogenes. Glucose metabolism in the bacteria generates gas pressure that actively pushes encapsulated drugs up to 1000 um into the skin, more than 200% deeper than static MNs. In a psoriasis animal model, this active transport improved therapeutic outcomes [67]. The strategy looks attractive for refractory plaques, yet living-microorganism systems still leave biosafety and sterilization questions unanswered. Several specific concerns arise with living-microorganism MN systems: residual bacterial viability and endotoxin contamination, potential colonization or translocation of the live cargo beyond the insertion site, unintended host immune activation against bacterial antigens, batch-to-batch variability of gas generation, and the difficulty of terminal sterilization without inactivating the bacteria [68]. For regulatory and clinical development, we suggest that live-engineered variants be replaced or supplemented by inactivated bacterial ghosts, lyophilized gas-generating chemical systems, or enzyme cascades that achieve the same deep-penetration effect without viable organisms; any residual live-bacteria approach would require sterility, biodistribution, immunogenicity, and environmental-release assessment in Good Laboratory Practice (GLP) studies before first-in-human testing.

Other designs aim at positioning accuracy and delivery efficiency rather than depth. Rotation microneedles modeled on honeybee stingers deform the skin less and hold the needle in place more precisely for sensing and delivery [69]. Negative-pressure MN syringes, derived from the old practice of fire cupping, combine suction with berberine-based nanocarrier delivery to ease drug passage through thickened stratum corneum [20]. Optical MN patches scatter near-infrared light in situ, triggering photothermal and photodynamic therapy within the basal layer and sidestepping the light attenuation typical of deeply pigmented or thickened skin [70]. Hollow MN arrays loaded with teriflunomide emulsomes have likewise enabled minimally invasive intradermal therapy [71]. Table 1 outlines the engineering characteristics and translational trade-offs of these platform families.

## 3. Nanovaccine Architectures and Targeting Strategies

**Figure 3:**
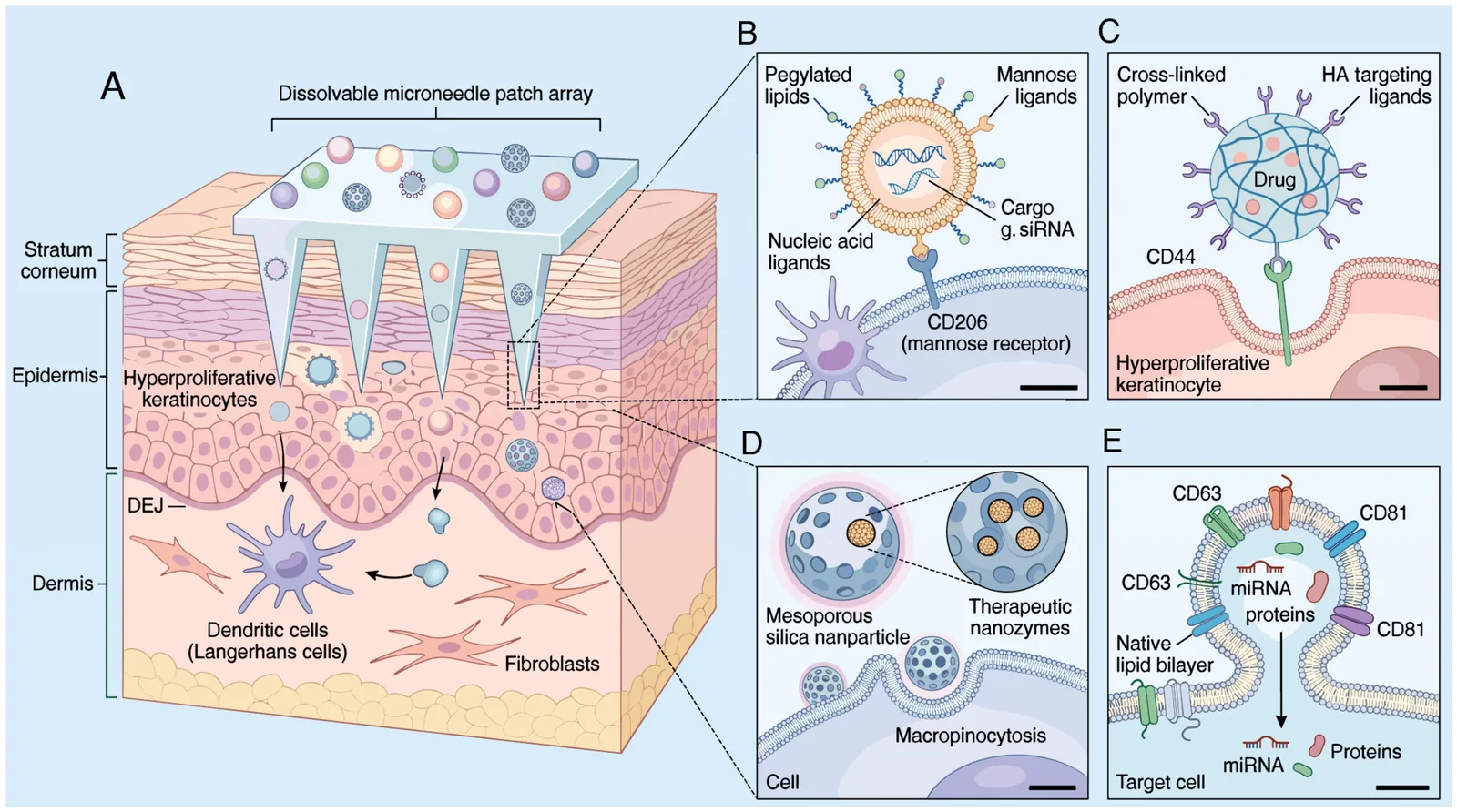
Schematic overview of dissolvable microneedle patch arrays and targeted nanocarrier architectures for tolerogenic immunotherapy in psoriasis. (**A**) Cross-sectional illustration of the skin showing a dissolvable microneedle patch array penetrating through the hyperkeratotic stratum corneum, epidermis, and dermal-epidermal junction (DEJ) into the dermis. The loaded nanocarriers (liposomes, nanoparticles) are released into the dermal tissue, where they interact with hyperproliferative keratinocytes, dermal fibroblasts, and dendritic cells (Langerhans cells) to initiate immune modulation. (**B**) Detailed structure of a lipid-based nanocarrier, including liposome, modified with pegylated lipids and mannose ligands. The cargo (g.siRNA) is encapsulated within the core. The mannose ligands specifically bind to CD206 (mannose receptor) expressed on dendritic cells, facilitating receptor-mediated endocytosis and targeted delivery of tolerogenic signals. (**C**) Illustration of a polymeric nanocarrier constructed from a cross-linked polymer and functionalized with hyaluronic acid (HA) targeting ligands. The drug payload is encapsulated within the matrix. The HA ligands bind to CD44 receptors, which are overexpressed on hyperproliferative keratinocytes, enabling targeted drug delivery to these pathogenic cells. (**D**) Depiction of a mesoporous silica nanoparticle (MSN) loaded with therapeutic nanozymes, including cerium oxide. This inorganic carrier enters target cells via macropinocytosis, providing a high-loading-capacity platform for enzyme-mimetic antioxidant therapies. (**E**) Schematic of a biomimetic nanocarrier based on extracellular vesicles (EVs) or exosomes. These vesicles are derived from native lipid bilayers, presenting surface markers such as CD63 and CD81. They shuttle endogenous cargo (miRNA and proteins) into target cells, influencing cellular pathways and regulating gene expression to support immune tolerance. Scale bar: 100 nm.

Microneedles cross the stratum corneum, but the nanocarrier adds more. It shields the cargo, holds it in the tissue longer, and guides it toward particular cells [42,43,44]. Nanocarrier technology has been extensively applied in transdermal delivery for cosmetic and therapeutic applications, providing a versatile platform for skin-targeted treatments [74]. Four carrier families dominate current work, and their surface ligands steer payloads to defined cutaneous cell populations at the dermal-epidermal junction (Figure 3).

### 3.1. Lipid-Based Nanocarriers: Elastic Liposomes, Ethosomes, and Nanostructured Lipid Carriers

Edge activators or ethanol make elastic (ultradeformable) liposomes and ethosomes more fluid, letting them travel deep through MN-created microchannels and improving cutaneous delivery of anti-inflammatory actives [75,76]. Hyaluronan-modified ethosomes build on CD44 overexpression to raise skin accumulation, as demonstrated with curcumin in psoriatic models [56]. Compared with conventional liposomes, nanostructured lipid carriers (NLCs) and solid lipid nanoparticles (SLNs) carry larger drug loads and remain more stable, and both have been extensively examined for skin applications [77,78]. Lipid-based systems, spanning NLCs, elastic vesicles, and spanlastics, have delivered apremilast, tacrolimus, resveratrol, and combinatorial siRNA cargoes in psoriasis studies [79,80,81,82]. Eugenol-loaded lipid nanoparticle hydrogels likewise relieved psoriasis-like skin lesions in preclinical work [83]. Berberine-loaded liposomes embedded in hydrogel microneedles improved permeability across the thickened epidermis and treated psoriasis-like lesions effectively [28], while MTX-bearing hyaluronan-modified liposomes inside dissolving microneedles enhanced intradermal delivery and limited systemic exposure [50].

### 3.2. Polymeric, Inorganic, and Nanozyme Carriers

Chitosan, polypeptide, and poly(lactic-co-glycolic acid) are common building blocks for biodegradable polymeric nanoparticles and nanogels, which combine tunable degradation with versatile surface chemistry [12,16,42,84]. Gentiopicroside-loaded chitosan nanoparticles checked TNF-alpha-driven keratinocyte proliferation and inflammation [84,85], and a polypeptide platform that exploits the disease-specific protein corona (LL37, S100A8/S100A9) co-delivered MTX and TNF-alpha siRNA to inflamed psoriatic skin [12]. Polymeric nanogels, valued for high water content, deformability, and stimulus-responsiveness, are being developed for topical inflammatory skin disease therapy [42,86], and high-drug-loading nanoparticle gels now deliver apremilast topically despite the thickened stratum corneum [29]. ROS-sensitive polyphenol nanoparticles paired with polysaccharide-based systems show how carrier design can handle penetration and oxidative stress at once [32]. Inorganic and enzyme-mimetic carriers add catalytic functions. Mesoporous silica nanoparticles (MSNs) carry large drug loads and can be engineered with dual pH/ROS responsiveness for anti-inflammatory therapy [87]. Zinc-doped MSNs loaded with betamethasone dipropionate, integrated into MN patches, improved transdermal delivery, favored M2 macrophage polarization, and outperformed the marketed cream on anti-inflammatory and anti-pruritic endpoints [88]. Metal-phenolic nanozymes, celastrol-loaded versions included, and mitochondria-targeted cerium oxide nanozyme systems have been combined with microneedles or thermoresponsive hydrogels to scavenge ROS, modulate the ROS/mTOR/HIF-1alpha axis, and curb oxidative damage in psoriatic skin [89,90,91]. Ceria nanoparticle-loaded double-network hydrogels similarly pair antioxidant activity with sustained corticosteroid release [92].

### 3.3. Biomimetic Extracellular Vesicles and Membrane-Camouflaged Platforms

Extracellular vesicles (EVs) and exosomes are natural lipid-bilayer nanoparticles loaded with endogenous proteins, lipids, and microRNAs. They are increasingly seen as both disease mediators and therapeutic candidates in skin inflammation [11,43]. Plant-derived vesicle-like particles are an especially convenient source. Perilla frutescens leaf-derived extracellular vesicle-like particles (EVPs) carrying pab-miR-396a-5p relieved psoriasis symptoms in prevention and treatment settings when given in hydrogels, with better retention in lesional skin [93]. Natural killer (NK) cell-derived EVs with anti-inflammatory activity have been delivered by microneedles and stabilized for storage, addressing the invasive delivery and stability problems that limit systemic EV therapy [94]. Membrane-camouflaged platforms extend the same idea to synthetic cores. Zhou developed a microneedle delivery platform integrated with Staphylococcus epidermidis-derived extracellular vesicle-based nanoantibiotics, providing a direct example of MN-EV combination for cutaneous therapy [95]. Keratinocyte-derived apoptotic nanovesicles expose phosphatidylserine as an eat-me signal that reroutes calcipotriol to DCs and reprograms their function, imitating natural efferocytosis [21]. Macrophage membrane-camouflaged nanoparticles loaded with etomoxir, delivered through a porous polydopamine MN patch, neutralize several inflammatory cytokines and target relapse-associated CD8 TRM cells, attacking the source of post-treatment recurrence [26]. Cell membrane vesicles engineered as IL-17RA decoys and delivered via MNs block engagement of multiple IL-17 ligands at once, an approach that mirrors and refines the systemic IL-17RA antibody strategy [13]. From a safety perspective, biomimetic EV systems require careful control of their biological cargo. Because EVs carry endogenous proteins, lipids, and nucleic acids, batch-to-batch heterogeneity, the possibility of transferring pro-inflammatory or oncogenic cargo, unintended immune activation, and immunogenicity upon repeated administration must be characterized [96,97]. Critical quality attributes should include particle-size distribution, surface-marker profiles, nucleic-acid content, sterility, and pyrogenicity, together with biodistribution and clearance studies, before clinical translation.

### 3.4. Pure-Drug Nanocrystals

Carrier-free nanocrystals give poorly soluble small molecules a way forward. They raise saturation, solubility and dissolution velocity without the toxicity or loading limits of synthetic carriers [49,98,99]. Tacrolimus nanocrystals (~260 nm) dispersed in an HA-based MN patch crossed the thickened epidermis and reached much higher cutaneous retention than the commercial ointment, with negligible systemic exposure [49]. MTX nanocrystals combined with dissolving MN arrays gave localized, sustained intradermal delivery in preclinical models [51], and apremilast nanocrystals, as novel topical formulations or nanocrystal-based gels, improved skin deposition and suppressed imiquimod-induced psoriatic inflammation [98,100]. Tetrandrine nanocrystals went further, promoting immunosuppressive CD4^+^FOXP3^+^ Tregs through the mTNF-TNFR2 pathway and tying a formulation choice directly to tolerance-relevant cellular outcomes [99]. Table 2 summarizes the physicochemical properties and translational challenges of the main nanovaccine carrier families.

## 4. Advanced Therapeutic Vaccine Paradigms and Multidimensional Intervention Strategies

Psoriasis rarely yields to a single agent, because the disease combines acute inflammation, chronic oxidative stress, neurogenic itch, and memory T cell-driven relapse [36,45,103]. Microneedle-nanovaccine platforms therefore increasingly bring together nucleic-acid or gene-editing payloads, photodynamic modalities, spatiotemporally programmed co-delivery, and interventions aimed at non-immune compartments, including the cutaneous microbiota and microvasculature.

### 4.1. Nucleic Acid and Gene-Editing Therapeutic Vaccines

Nucleic acid therapeutics can act at the transcriptional or genomic level, but systemic delivery suffers from rapid enzymatic degradation, poor cellular uptake, and delivery-system-related inflammation [33,104,105,106]. siRNA-based nanotherapeutics have accordingly been developed for topical and transdermal silencing of psoriatic mediators. TNF-alpha siRNA has traveled through photochemical-internalization-assisted nanoparticles, fusogenic lipid nanoparticles, and NLCs co-loaded with tacrolimus or other actives [80,107,108]. Erlotinib/IL-36alpha siRNA co-delivery, MTX/siRNA-TNFalpha combinations, and anti-inflammatory LNPs that silence disease-relevant genes all reduced psoriatic inflammation in imiquimod models [12,106,109]. Microneedles enable direct intradermal siRNA delivery. MN arrays delivered siRNA transdermally in proof-of-concept work [110], laser-assisted nanocarrier delivery achieved cutaneous siRNA targeting in psoriasis models [111], and biomimetic nanovesicle-loaded MNs carried PCSK9 siRNA to adjust lipid-metabolism-driven keratinocyte hyperproliferation and immune dysregulation [112]. A biomineralized nanoparticle-incorporated MN system recently focused on SLC31A1, linking cuproptosis and ferroptosis in psoriatic keratinocytes and widening the set of druggable metabolic nodes [113]. Dendritic lipopeptide-based transdermal siRNA systems that silence STAT3 in cutaneous immune cells illustrate the cell-type-specific silencing that tolerance-oriented therapy needs [27]. MicroRNA-based strategies add an epigenetic layer. Disease-specific microRNA profiles have been mapped in lesional psoriatic skin, and topical delivery of microRNA-210 antisense or anti-inflammatory microRNA therapeutics changed keratinocyte proliferation and immune balance in animal models [6,114,115,116]. Topical microRNA-directed therapy is still an active translational area, and microneedle-based sampling patches now detect psoriatic microRNA biomarkers in interstitial fluid [117,118]. mRNA and CRISPR-based gene editing sit at the frontier of cutaneous vaccination. A lipoic acid-based ionizable lipid library was built to merge antioxidant activity with thiol-mediated uptake, and the lead LNP formulation delivered Cas9 mRNA together with CD93 sgRNA locally in a murine psoriasis model, achieving effective CD93 genome editing and inhibition of the CD93-p38 MAPK-AKT-SMAD2/3 pathway [119]. Microneedle-assisted CRISPR-Cas9 delivery has also enabled transdermal co-delivery of Cas9 ribonucleoprotein targeting NLRP3 alongside dexamethasone nanoparticles, resolving inflammatory skin lesions preclinically [120], and dual-responsive nanocarriers have pushed cytosolic protein and Cas9 delivery forward [121]. Reviews of CRISPR-guided dermatologic therapy and biophysical carrier design lay the groundwork for clinical translation, although no CRISPR-based trial for cutaneous disease has begun [122,123]. By deleting pro-inflammatory genes in keratinocytes or immune cells, gene-editing nanovaccines could, in principle, change the disease course rather than manage symptoms [124]. Before gene-editing nanovaccines can be considered for psoriasis, several safety and regulatory questions must be answered. CRISPR-Cas9 can produce off-target edits, and immune responses against the Cas9 protein or the delivery vector may neutralize repeated dosing or trigger inflammatory reactions [125]. Because psoriasis is a chronic but generally non-life-threatening disease, the risk-benefit threshold for permanent genomic modification is high [126]; somatic-cell genome editing of this type will require rigorous on-/off-target analysis, long-term genotoxicity assessment, and early regulatory engagement on classification, monitoring, and reversibility [127].

### 4.2. Stimuli-Responsive Smart Release and Photodynamic Combination Therapies

Multi-responsive, size-switchable nanoparticles delivered by MNs confront a basic paradox. Particles large enough to remain in the dermis are poorly internalized by keratinocytes, while small particles enter cells easily but clear quickly. A microneedle platform using multi-responsive, keratinocyte-targeted nanoparticles that shift from larger dermal-retentive particles to ultra-small fragments in the mildly acidic, ROS-rich psoriatic microenvironment improved intracellular delivery to hyperproliferative keratinocytes [30]. Biomineralized in situ catalytic nanoreactors integrated into MN patches have also been reported for on-demand immunomodulation of inflammatory skin [128]. Photodynamic therapy (PDT) attracts interest for psoriasis because it is non-invasive and selective, yet translation is limited by weak cutaneous permeability, light attenuation, and pro-oxidative effects that can undercut the benefit [70,72,129]. Shield microneedles loaded with biphasic-delivery liposomes containing 5-aminolevulinic acid and ginsenoside Rg3 tackle the oxidation/immunity axis by switching on antioxidant enzyme systems during ALA-PDT [129]. Hollow MN-assisted delivery of hypericin-loaded NLCs combined with PDT improved drug delivery and antipsoriatic activity in imiquimod-treated mice [72]. Oxygen-boosted dual-section MN patches co-deliver a photosensitizer with an oxygen generator, sodium percarbonate, relieving lesion hypoxia and amplifying PDT efficacy, while a ROS-responsive coating controls drug activation in space and time [73,130]. Precision-engineered dissolving MNs made by projection microstereolithography enable green-light-activated chemo-photodynamic combination therapy with tight control over drug activation [131]. Mechanistically, a cerium-doped polyoxometalate nanoplatform that neutralizes pathogenic double-stranded RNA and ROS together suppressed type I interferon responses and cutaneous inflammation, a reminder that inorganic nanomaterials can engage both the nucleic-acid and oxidative arms of psoriasis pathology [132].

### 4.3. Cascaded Co-Delivery and Depletion of Relapse-Associated Memory T Cells

Pathogenic CD8 TRM cells lingering in resolved skin explain much of the high relapse rates observed after treatment withdrawal in some studies [26]. Because conventional drugs leave this memory pool intact, MN systems are being designed to co-deliver acute anti-inflammatory and relapse-preventive cargoes with separate release kinetics. The self-locking deucravacitinib/calcipotriol patch and the ROS-responsive MTX/CeO_2_ bilayer MN combine rapid anti-inflammatory action with sustained antiproliferative action [60], illustrating how cascaded co-delivery can be engineered into a single platform. A dual-layer MN combining methotrexate-zinc (MTX-Zn) with difelikefalin (DFK) joins sustained anti-inflammatory and antibacterial effects to rapid antipruritic action, breaking the itch-scratch-inflammation cycle [133]. Going straight for the TRM compartment, Huang et al. screened a macrophage membrane that recognizes multiple psoriatic inflammatory factors and used a porous polydopamine MN patch to co-deliver the membrane with etomoxir, a metabolic inhibitor that disrupts the metabolic fitness of memory T cells [26]. The combination, cytokine decoying plus metabolic disruption of TRM cells, cut relapse-driving immune memory while suppressing acute inflammation, ranking among the first MN-based attempts to tackle disease recurrence mechanistically [26]. Combined with transdermal IL-17 inhibitors that block recurrence after treatment ends, including the cyclic tetradecapeptide-loaded lipopeptide liposomes that sustained IL-17 inhibition and prevented recurrence [14], these platforms point toward durable remission rather than temporary closure.

### 4.4. Cutaneous Microbiota Modulation and Endothelial Glycocalyx Repair

Beyond immune cells, two targets have begun to attract attention. The first is the cutaneous microbiome. Its exchanges with the skin immune system help determine homeostasis and disease, and microbial niches around hair follicles appear especially influential [134]. Direct proof that manipulating the microbiota can treat psoriasis remains scarce, but MN-delivered nanogels and antimicrobial nanoparticles that reshape the lesional microbial environment offer a plausible avenue [41,134]. Supporting the concept, extracellular vesicles derived from commensal skin microbiota have been shown to alleviate cutaneous inflammation in atopic dermatitis by re-establishing skin homeostasis [135]. The second target is the vascular endothelium, which is now viewed as a gatekeeper for immune cell entry into psoriatic skin. Ou et al. described a pathogenic COX2 endothelial cell subpopulation distinguished by elevated P-selectin expression, oxidative stress, and MMP9-mediated glycocalyx degradation; they then built inflammation-triggered nanodrug MN patches (P@Meb-MNs) loaded with the FDA-approved anthelmintic mebendazole to shut down the ROS-COX2-MMP9 axis, restore endothelial glycocalyx integrity, and prevent leukocyte extravasation into the skin [136]. Endothelial-directed MN therapy thus homes in on a non-immune barrier whose failure feeds inflammatory infiltration. These non-immune interventions are complementary to, rather than substitutes for, tolerogenic vaccination: by restoring microbial homeostasis and endothelial barrier integrity, they reduce the inflammatory milieu in which autoreactive effector cells persist and thereby create a more permissive environment for antigen-specific tolerance induction.

## 5. Immunomodulatory Networks of Tolerogenic Nanovaccines in the Psoriatic Skin Microenvironment

**Figure 4:**
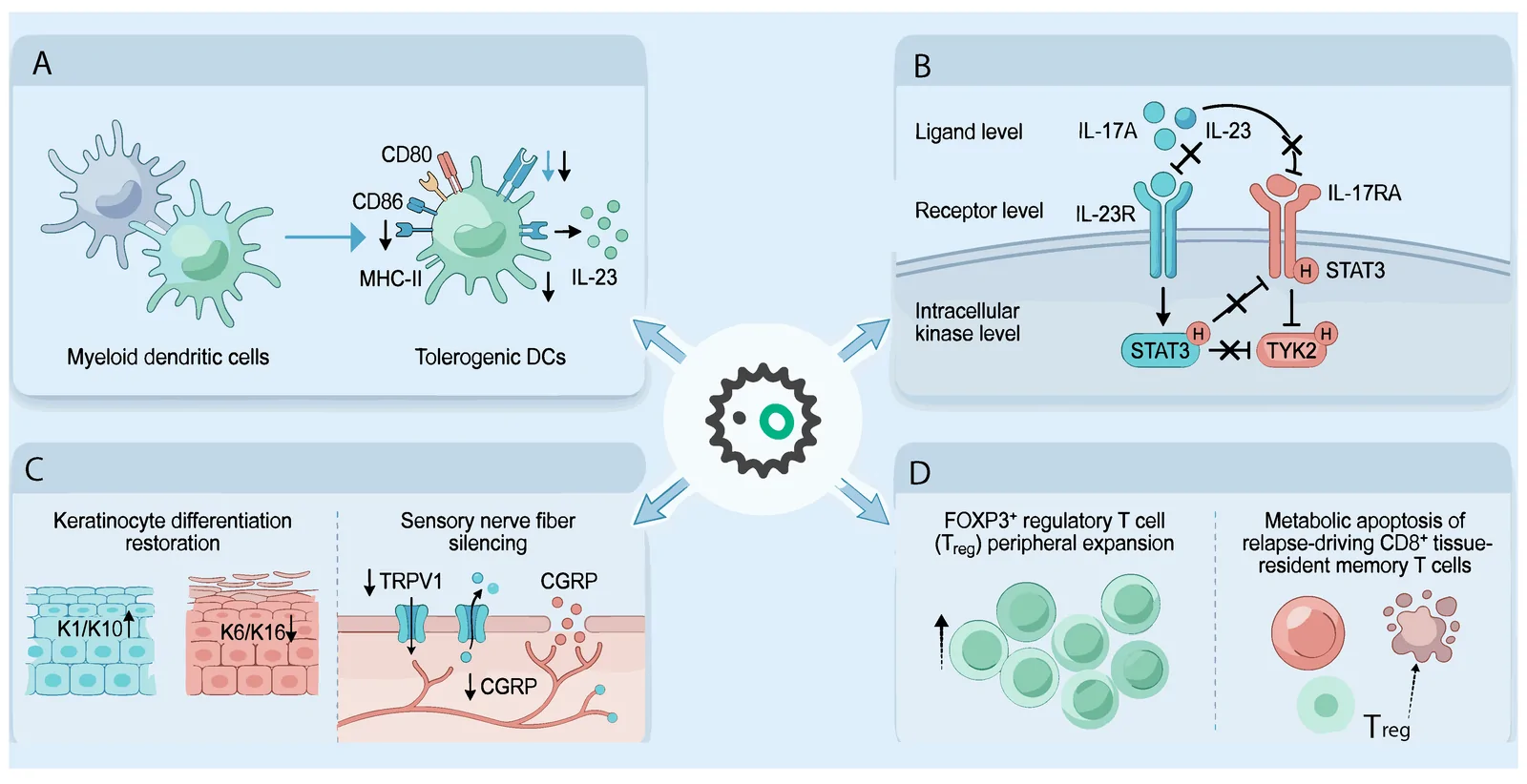
Integrated immunomodulatory networks of transdermal tolerogenic nanovaccines. Mechanistic map detailing central tolerogenic nanovaccine-mediated immune re-education across four interconnected pathways: (**A**) DC tolerogenization through downregulation of surface co-stimulatory molecules (CD80/CD86, MHC-II) and reduced IL-23 secretion; (**B**) Multi-level blockade of the IL-23/IL-17 axis at receptor, ligand, and intracellular STAT3/TYK2 kinase levels; (**C**) Keratinocyte differentiation normalization (K1/K10 upregulation vs. K6/K16 suppression) and neuro-sensory itch relief via the TRPV1/CGRP nerve axis; (**D**) FOXP3^+^ Treg cell expansion alongside metabolic apoptosis of relapse-driving CD8^+^ TRM memory T cells, culminating in long-term cutaneous tolerance and relapse prevention. In this figure, solid arrows denote mechanisms demonstrated in the integrated microneedle-nanocarrier studies cited in the text, whereas dashed arrows denote proposed mechanisms extrapolated from separate fields that remain to be demonstrated with the integrated platforms.

Cutaneous tolerogenic nanovaccines are compelling because they can retune several immune-cell types and signaling networks at once, shifting the balance from inflammation toward tolerance. Figure 4 maps the principal molecular pathways discussed below.

### 5.1. Induction and Reorganization of Tolerogenic Dendritic Cells

DCs both initiate and sustain psoriatic inflammation through antigen presentation and cytokine release, which makes them the obvious first target for tolerance induction [17,21,22]. Nanocarriers can steer tolerogenic cargo toward DCs specifically. Mannosylated liposomes carrying celastrol boosted DC uptake and induced DC tolerance in imiquimod-induced psoriasis models [22], and keratinocyte-derived apoptotic nanovesicles bearing an eat-me phosphatidylserine signal rerouted calcipotriol to cutaneous DCs, converting their phenotype from pro-inflammatory to regulatory [21]. These approaches conceptually imitate the way DCs normally clear apoptotic keratinocytes, a homeostatic process disrupted in psoriasis [11,21]. Reviews of tolerogenic nano-formulations for autoimmune disease emphasize that carrier composition, antigen context, and the absence of inflammatory danger signals together determine whether DCs adopt a tolerogenic (tDC) phenotype [17,18]. In skin, tDCs express fewer co-stimulatory molecules and more IL-10 and TGF-beta, directing differentiation toward FOXP3 Tregs rather than effector T cells [22,44].

### 5.2. Blockade of Th17/Th1 Effector Differentiation and Pro-Inflammatory Cascades

The IL-23/IL-17 axis is the dominant effector pathway in plaque psoriasis, and MN-delivered vaccines can interrupt it on several levels [2,13,14]. At the receptor, IL-17RA decoy vesicles delivered through MNs sequester multiple IL-17 ligands at once, broadly damping downstream signaling without the systemic IL-17C-elevation worries attached to anti-IL-17RA antibodies [13]. At the ligand level, transdermal lipopeptide liposomes carrying a metabolically stable cyclic tetradecapeptide inhibitor of IL-17 sustained cytokine inhibition and prevented recurrence in psoriasis models [14], and anti-IL-17A aptamer-gold nanoparticle conjugates improved anti-inflammatory efficacy in imiquimod-induced disease [15]. At the signaling level, topical deucravacitinib-based nanotherapeutics dampen the TYK2-dependent arm of the IL-23/IL-17 pathway [16,63,137]. siRNA-mediated silencing of IL-17 pathway components and STAT3 further shows that nucleic-acid nanovaccines can act upstream of cytokine secretion [27,106,113]. The distinction between conventional immunosuppression and targeted systemic therapy is clinically important: traditional systemic agents such as methotrexate, cyclosporine, and acitretin suppress immunity broadly, whereas modern biologics and oral targeted drugs act on defined pathways, including TNF-alpha, IL-23/IL-17, and TYK2, with different efficacy, safety, and monitoring profiles [24,25]. A recent review of systemic psoriasis treatment clearly distinguishes conventional immunosuppressants, including methotrexate, cyclosporine, and acitretin, from targeted systemic therapies, including both biologics and oral small molecules, with respect to efficacy, safety, monitoring, and treatment goals [138]; none of these modalities is designed to restore antigen-specific tolerance, which remains the rationale for the tolerogenic strategies reviewed here.

### 5.3. Correction of Keratinocyte Hyperproliferation and Suppression of Neurogenic Itch

Keratinocyte hyperproliferation and failed differentiation are the histologic signatures of psoriasis, driven by IL-17, TNF-alpha, and oxidative stress [6,36,84,139]. MN platforms deliver anti-proliferative agents directly to the basal epidermis. These agents include MTX, tacrolimus, calcipotriol, glucocorticoids, and natural products, curcumin, berberine, baicalin, and shikonin among them, normalizing proliferation and differentiation while avoiding systemic exposure [28,56,57,63,64,140,141]. Antioxidant nanozyme systems take on the oxidative component of keratinocyte dysfunction [90,91,92], and compensatory ROS-management strategies built on DNA nanostructures have been suggested to avoid the collateral harm of total ROS depletion. Pruritus is a heavy clinical burden, and several MN systems carry fast-acting antipruritic cargoes. The MTX-Zn/DFK dual-layer patch pairs sustained anti-inflammatory activity with rapid suppression of neurogenic itch [133], and zinc-doped MSN/betamethasone MN therapy beats the marketed cream on anti-pruritic outcomes [88]. Clinical comparisons of topical MTX hydrogel formulations for scalp psoriasis confirm that itch control is a meaningful, measurable endpoint of topical therapy [142]. Together these observations argue that a single MN patch combining anti-inflammatory, antiproliferative, and antipruritic functions can meet the symptom triad behind patient suffering and adherence failure. Beyond objective disease scores, patient-reported outcomes (PROs) matter for a self-administered transdermal platform [143,144]. Psoriasis profoundly affects health-related quality of life, treatment satisfaction, and adherence [145,146], and PROs especially the Dermatology Life Quality Index (DLQI), pruritus visual analogue scales, treatment-satisfaction questionnaires, and adherence diaries should be incorporated into early clinical studies of MN-based therapies [144]. Usability and acceptability endpoints, including pain, application time, wearing comfort, and ease of removal, are particularly relevant for MN patches because they determine whether patients sustain treatment [147]. We recommend that future trial designs include both clinician-reported (PASI, PGA) and patient-reported endpoints [144,145].

### 5.4. Expansion of Regulatory T Cells and Re-Establishment of Antigen-Specific Tolerance

Tregs are numerically and functionally deficient in psoriatic skin, and restoring them is central to durable disease control [18,44,99]. Nanovaccine platforms expand Tregs through several routes. Tetrandrine nanocrystals promoted immunosuppressive CD4+FOXP3+ Tregs via the mTNF-TNFR2 pathway while clearing the solubility and penetration hurdles that limit topical delivery [99]. Adoptive Treg therapy takes a more direct route. Perforated microneedles with a spacious encapsulation cavity supported Treg survival and migration and enabled localized adoptive Treg therapy of psoriasis, while an enzyme-degradable metabolic-intervention component countered the phenotypic instability and functional loss that have stalled systemic Treg therapy [23]. Vitamin D receptor signaling, engaged by calcipotriol-containing MN systems, further ties keratinocyte-directed therapy to tolerogenic immune modulation [4,148,149]. Combined with tDC-inducing nanocarriers [21,22], these strategies seek to restore the antigen-specific tolerance that conventional immunosuppressants cannot deliver, preventing relapse instead of merely quieting flares.

Tolerogenic Dendritic Cell Phenotypes and Treg Subsets in Psoriasis. tDCs express low MHC-II/CD80/CD86, reduced IL-12/IL-23/IL-6, and high IL-10/TGF-beta/IDO [150]. When they present self-antigens amid weak co-stimulation, tDCs push conventional T cells toward anergy or apoptosis and favor FOXP3 Treg expansion [150,151]. The relationship cuts both ways, because Tregs reinforce the tDC phenotype through CTLA-4 and IL-10/TGF-beta [152]. The Treg compartment itself is far from uniform. Resting (CD45RA FOXP3 low) and activated (CD45RA-FOXP3 high) Tregs coexist [153], as do FOXP3- Tr1 cells (IL-10LAG-3CD49b), whose numbers fall in psoriatic blood and correlate inversely with PASI [151,154], and iTr35 cells that secrete IL-35 [155]. A critical caveat is that under IL-23/IL-6/IL-21, skin Tregs can lose FOXP3, upregulate RORγt, and assume a Th17-like identity, a plasticity that feeds local inflammation [151,156]. This instability, traceable to impaired TSDR demethylation and weak IL-2 signaling, constitutes a key barrier to tolerogenic vaccines [157]. Advanced MN-nanovaccine platforms therefore aim to do more than expand Tregs; they try to stabilize the phenotype and resist plasticity within the psoriatic microenvironment.

### 5.5. Antigen Selection for Psoriasis Tolerogenic Vaccines

A tolerogenic vaccine can be no more specific than the antigen it carries, and psoriasis still lacks a firmly established set of autoantigens. Several candidates have been proposed, from LL-37-nucleic acid complexes and S100 proteins to antigens tied to aberrant epidermal differentiation [158,159,160]. LL-37 is one of the best studied. It binds self-DNA/RNA, activates plasmacytoid DCs, and is recognized by T cells infiltrating psoriatic skin [158,160]. The central difficulty is that no consensus psoriasis autoantigen has been confirmed, so any choice of antigen must balance tolerance induction against the risk of bystander immunity [19,161]. Particle-based inverse vaccination, which presents antigens in a tolerogenic context, is being explored across autoimmune diseases and could serve as a blueprint for psoriasis-specific antigen discovery and validation [161,162,163].

How an autoantigen is delivered shapes the tolerogenic outcome. Naked peptides are cleared quickly and can prime effector responses under inflammatory conditions [19]. Nanoparticle formulations, by contrast, can pair autoantigens with tolerogenic adjuvants, rapamycin, TGF-beta, or aryl hydrocarbon receptor ligands, steering tDC differentiation and antigen-specific Treg expansion [163]. mRNA-encoded autoantigens in lipid nanoparticles represent an emerging alternative. They allow endogenous antigen production while nucleoside modifications dampen innate immune activation [44].

## 6. Translational Bottlenecks, Safety, and Regulatory Science

Moving MN-nanovaccine platforms from animal models into clinical practice demands coordinated advances in mechanics, manufacturing, safety, and regulation [40,41,45]. Beyond these technical and regulatory considerations, patient-reported outcomes (PROs) and treatment acceptability, including pain upon application, wearing comfort, ease of removal, and impact on daily activities, represent an equally critical translational pillar, as they determine real-world adherence and ultimately the clinical success of a self-administered patch platform.

### 6.1. Mechanical Performance, Insertion Safety, and Microchannel Closure

Psoriatic plaques are thickened and mechanically uneven, which places demanding requirements on MN insertion [34,35,45]. Needle geometry, length, and tip sharpness must be tuned so needles cross the stratum corneum without fracturing or hurting. 3D printing now permits systematic variation in geometries, including square pyramidal, conical, and obelisk shapes, and lengths from 400 to 800 μm or more, to improve insertion into compromised skin [34,47]. Rotation-based insertion reduces skin deformation and sharpens positioning accuracy [69], and implantable tip-accumulated MN designs have been developed for long-term psoriasis care with fewer doses [164]. Polymer residues and microchannel closure kinetics influence both efficacy and infection risk, so removability, as with HFMNs, and controlled closure remain active design considerations [40,59]. Ex vivo and formulation studies on human skin, MN/iontophoresis combinations for MTX and hollow MN delivery of JAK inhibitors included, supply the mechanistic basis for choosing these parameters [35,165].

### 6.2. Scalable Manufacturing, Quality Control, and Sterilization

Scaling multicomponent nanovaccine-MN products industrially calls for quality-by-design (QbD) approaches that standardize tip geometry, drug loading, and particle-size distribution [34,41,47]. Continuous liquid interface production and projection microstereolithography allow high-precision MN fabrication with reproducible geometry [34,131], and 3D-printed MN arrays are positioned as a platform for personalized dermal therapeutics [47]. Sterilization poses its own bottleneck. Heat and ionizing radiation can destroy nucleic acids, proteins, and polymeric matrices, so aseptic molding, low-temperature processes, and formulation-based stabilization, lyophilization and hydrogel encapsulation of EVs among them, are under investigation [47,93,94]. Systematic reviews of dissolving MN use in skin conditions conclude that feasibility, tolerability, and manufacturability all need proof before regulatory engagement [45,47]. For regulatory and quality-by-design purposes, the dose, loading capacity, and release kinetics of both the nanocarrier and the antigen or cargo must be standardized. Critical quality attributes should include the antigen/nanocarrier dose per patch, antigen loading capacity and encapsulation efficiency, particle size and polydispersity, and the release profile measured in biorelevant in vitro release tests, together with acceptance criteria defined a priori. In vitro-in vivo correlation studies will be needed to support batch consistency and regulatory approval.

### 6.3. Regulatory Pathways for Drug-Device Combinations and Clinical Trial Design

MN-nanovaccine systems are inherently drug-device combination products, so their regulatory assessment must cover the active pharmaceutical ingredient, its pharmacokinetics, pharmacodynamics, and immunotoxicity, and the delivery device, its mechanical safety, skin irritation, sensitization, and microchannel closure [37,38,45]. Reviews of MN technology in psoriasis management note that early-phase clinical data are still thin and that trial design must accommodate plaque-thickness heterogeneity, lesion-site variability, and patient-reported outcomes, pruritus and adherence included. Current clinical evidence for MN-based psoriasis therapy is largely confined to proof-of-concept and small comparative studies of conventional actives [40,45], and, to the best of our knowledge, no MN-delivered tolerogenic vaccine has entered clinical testing. Combination-product pathways in dermatology are therefore best handled conservatively, with phased evaluation of device performance, local tolerability, and pharmacokinetics before efficacy claims [36,45,46]. Engagement with regulatory agencies on classification, chemistry-manufacturing-controls, and clinical endpoints for MN-based vaccines will be necessary as the field matures. Contextual comparison with current standard-of-care biologics is important for positioning this technology. Anti-IL-17, anti-IL-23, and anti-TNF-alpha biologics achieve high and durable skin clearance in many patients but require systemic administration, carry class-specific safety signals, including mucocutaneous candidiasis with IL-17 blockade, and generally require continuous treatment because they do not re-establish tolerance [24,25]. MN-delivered tolerogenic nanovaccines are at an earlier stage of development and currently lack clinical efficacy data; their potential advantages, localized delivery, lower systemic exposure, and the possibility of durable, relapse-preventing immune re-education, remain hypotheses to be tested in translational and clinical studies. Cost-effectiveness cannot yet be assessed reliably and will depend on manufacturing scale, dose frequency, and the durability of response once clinical data become available (Table 3).

### 6.4. Immunological and Long-Term Safety Considerations

Beyond insertion mechanics and sterilization, the immunologic and long-term safety of MN-nanovaccine combinations requires dedicated evaluation. Nanocarriers and nucleic-acid payloads can themselves activate innate immune sensors, including Toll-like receptors and cGAS-STING, which could inadvertently prime rather than tolerate [166]; tolerance-inducing strategies must therefore be shown not to weaken protective immunity against pathogens, i.e., they should not induce systemic immunosuppression or tolerance to protective antigens. Additional risks include Treg instability and conversion to effector phenotypes [167]; infection through microchannels during the closure period [168]; nanoparticle persistence and accumulation in draining lymph nodes or organs [169]; and immunogenicity of repeated administration, including anti-drug/anti-carrier antibodies and hypersensitivity [170]. Preclinically, these risks should be assessed through local and systemic immunotoxicity panels, tissue biodistribution and clearance studies, and long-term toxicology in relevant species. Clinically, first-in-human protocols should include sentinel-dose safety evaluation; local tolerability and wound-healing assessments; monitoring of systemic immune parameters, including cytokines, lymphocyte subsets, and anti-drug antibodies; and long-term follow-up for infection, malignancy, and relapse dynamics [168].

## 7. Challenges and Future Perspectives

### 7.1. Limitations of Preclinical Models and Human-Relevant Validation

Most of the efficacy data reviewed here were generated in imiquimod-induced psoriasiform dermatitis in mice. This model recapitulates key inflammatory features of psoriasis, especially IL-23/IL-17-driven keratinocyte hyperproliferation and neutrophilic infiltration, but it is an acute, chemically induced model that does not fully reproduce the chronicity, genetic background, antigen-specific autoimmunity, or disease relapse of human psoriasis [171]. Consequently, reductions in skin inflammation in this model demonstrate anti-inflammatory activity but cannot by themselves establish immune tolerance. To strengthen translational relevance, future work should complement imiquimod models with human-relevant systems: human skin equivalents and organotypic 3D skin models [172], ex vivo human psoriatic skin explants, and mouse models with humanized immune compartments or antigen-driven psoriatic phenotypes [173], together with standardized readouts for antigen-specific Treg induction and relapse upon treatment withdrawal.

### 7.2. Open Questions and Near-Term Milestones

The barriers to clinical translation are not only engineering and regulatory; substantial conceptual and immunological uncertainties remain, above all the absence of a validated psoriasis autoantigen and the lack of direct clinical evidence for durable, antigen-specific tolerance induction. [40,45,47]. Reviews of MN technology in psoriasis management repeatedly observe that early-phase clinical data are scarce and that device performance, local tolerability, and pharmacokinetics should be assessed in phases before efficacy claims [40,45,46]. The near-term steps most likely to de-risk the field are standardized ex vivo penetration and irritation models using psoriatic human skin [34,35], first-in-human studies of MN-delivered conventional actives to confirm device safety [40,142], and mechanistic biomarker studies linking tDC/Treg induction to clinical response in plaque psoriasis [22,23,99]. Several questions stay open. The first is whether tDC and Treg responses seen preclinically become durable, antigen-specific tolerance in patients, which has not been tested. The antigenic identity of a psoriasis tolerogenic vaccine, be it self-peptide, LL-37-nucleic acid complexes, or disease-associated neoantigens, is likewise unresolved [12,21,23]. A second question concerns how much the stratum corneum barrier, the lesional microbiome, and the microvasculature each shape vaccination outcome, although endothelial glycocalyx repair and microbiota-directed interventions are emerging as workable targets [134,136]. Pairing tolerogenic vaccination with acute anti-inflammatory or antipruritic treatment raises further questions about dose, timing, and release kinetics, although existing bilayer and stimuli-responsive platforms provide a flexible starting point [60,63,133]. Regulatory endpoints for therapeutic vaccines in inflammatory skin disease are likewise unstandardized, and consistent classification of MN-nanovaccine combinations across agencies would speed up evaluation [37,38,45]. The answers to these questions will decide whether transdermal tolerogenic vaccination can become a realistic option for psoriasis and related inflammatory skin diseases.

Clinical data on microneedle platforms in psoriasis are limited but accumulating. In nail psoriasis, a randomized controlled trial reported that corticosteroid-loaded dissolvable microneedles performed as well as conventional topical therapy and caused no severe adverse events [174]. For tolerogenic vaccination, the most advanced preclinical candidate is a microneedle patch carrying regulatory T cell-derived exosomes, which lessened psoriatic inflammation in vivo [175]. Clinical experience with tolerogenic cell and vaccine approaches in other autoimmune diseases, from tolerogenic dendritic cell vaccines to antigen-specific immunotherapy, remains early; feasibility has been shown, but efficacy endpoints are still unresolved [176,177,178]. A sensible first-in-human program for MN-based tolerogenic vaccines in psoriasis would open with device safety and local tolerability, then incorporate pharmacokinetic and mechanistic biomarker endpoints, including tDC and Treg induction, before any efficacy claims [178].

## 8. Conclusions

The review demonstrates that the combined application of microneedles and nanocarriers offers a promising technological pathway for tolerogenic immunotherapy in psoriasis. These systems can breach the hyperkeratotic skin barrier and deliver immunomodulatory payloads in a targeted manner to the dermal-epidermal junction, where they reprogram the inflammatory microenvironment by inducing tolerogenic dendritic cells, expanding regulatory T cells, and blocking the IL-23/IL-17 effector axis. Preclinical studies have provided initial proof-of-concept for this strategy, yet no single platform has simultaneously achieved all of the anticipated effects, and most studies remain at the animal-experiment stage. Current bottlenecks include the lack of well-defined psoriasis autoantigens, the limitations of imiquimod-induced animal models in recapitulating human disease, and the absence of unified clinical and regulatory standards. If these conceptual, immunological, and engineering hurdles can be overcome in the future, microneedle-based tolerogenic vaccination holds promise as an effective and durable therapeutic strategy for psoriasis and other chronic inflammatory skin diseases characterized by the loss of cutaneous immune tolerance.

## Figures and Tables

**Table 1:** Engineering characteristics and translational comparison of microneedle transdermal vaccine delivery platforms for psoriasis.

Platform Type	Matrix/Composition & Release Mechanism	Advantages	Translational Limitations	Representative Systems	Level of Evidence	Refs
Dissolving MNs	HA, CMC, PVP, PVA, saccharide matrices; dissolve on interstitial-fluid uptake; rapid payload release to epidermis/dermis	Painless, no sharps waste; HA enables CD44-mediated targeting	Limited payload capacity; humidity-sensitive storage	MTX, tacrolimus nanocrystals, TNF-α antibody, calcipotriol nanosuspension; imiquimod (IMQ) mouse model; improved skin retention and reduced inflammation	In vitro/in vivo (preclinical)	[40,48,49,50]
Hydrogel-forming MNs	Crosslinked hydrophilic networks (GelMA, PMVE/MA, PEGDA); swell to form hydrogel conduits; reservoir-driven diffusion	Removed intact with zero polymer residue; suitable for sustained delivery	Requires backing reservoir; initial lag time	Phytochemicals and macromolecular payloads under investigation; in vitro and ex vivo human-skin permeation studies	In vitro/ex vivo (preclinical)	[41,59]
Stimuli-responsive MNs	ROS-labile matrices (PVA-TSPBA), MMP-cleavable crosslinkers; matrix degradation triggered by ROS, pH, or proteases	On-demand release matched to disease severity; reduced off-target exposure	Complex synthesis; batch-to-batch trigger variability	MTX/EGCG gel MN, MTX–CeO_2_ bilayer MN, deucravacitinib prodrug nanoassemblies; IMQ mouse model; prolonged drug levels and reduced IL-17/ROS	Preclinical (in vivo)	[31,60,61,62]
Bilayer/self-locking MNs	PVA inner tip + HAMA/GelMA outer sheath or backing; rapid tip dissolution plus swelling-mediated mechanical anchorage	Resists detachment on flaking plaques; spatiotemporal multi-drug release	Complex two-step molding; demanding tip mechanics	Deucravacitinib/calcipotriol self-locking MN, MTX/dexamethasone bilayer, methylprednisolone/upadacitinib-MSN bilayer; IMQ mouse model; dual anti-inflammatory/antiproliferative effects	Preclinical (in vivo)	[63,64,65]
Micro-engine MNs	Pneumatic patch with live Enterobacter aerogenes; bacterial gas generation actively propels payload > 200% deeper	Overcomes passive-diffusion limits; effective in thick plaques	Biosafety and sterilization concerns	Live E. aerogenes pneumatic MN; IMQ mouse model; deeper drug delivery and improved outcomes	Preclinical proof-of-concept	[67]
Hollow/optical MNs	Hollow needles; light-scattering or oxygen-generating designs; direct infusion or light/oxygen-enhanced activation	Enables photodynamic combinations; basal-layer activation	Device complexity; light source requirements	Teriflunomide emulsomes (hollow MN), hypericin-NLC + PDT, oxygen-boosted PDT MN; IMQ mouse model; improved permeation and PDT efficacy	Preclinical (in vivo)	[70,72,73]

**Table 2:** Physicochemical properties, delivery advantages, and translational challenges of primary tolerogenic nanovaccine carriers.

Carrier Type	Representative Payloads	Advantages in MN Platforms	Translational Limitations	Representative Systems	Level of Evidence	Refs
Elastic liposomes/ethosomes	Curcumin, berberine, 5-ALA	High deformability for deep penetration through MN channels	Leakage and aggregation in storage; thermal instability	Curcumin-loaded hyaluronan-modified ethosomes, berberine liposomes in hydrogel MNs; IMQ mouse model; improved skin accumulation and reduced lesions	Preclinical (in vivo)	[28,56,75]
NLCs/SLNs	Tacrolimus, apremilast, TNF-α siRNA	High payload; sustained release; skin occlusion	Limited surface functionalization; strict lipid control	Apremilast NLC, tacrolimus/TNF-α siRNA NLC; IMQ mouse model and in vitro studies; reduced inflammation	Preclinical (in vitro/in vivo)	[77,79,80,101]
Polymeric nanoparticles/nanogels	MTX, siRNA, natural products	Tunable degradation; versatile ligand conjugation	Solvent residues; potential contact sensitization	MTX/siRNA-TNFα corona nanoplatform, gentiopicroside chitosan NPs; IMQ mouse model and HaCaT cells; reduced TNF-α and keratinocyte proliferation	Preclinical (in vitro/in vivo)	[12,32,84,102]
Mesoporous silica/nanozymes	Betamethasone, celastrol, resveratrol	High loading; ROS scavenging; M2 polarization	Slow scaffold degradation; long-term accumulation data needed	Betamethasone zinc-doped MSN MN, celastrol nanozymes; IMQ mouse model; M2 polarization, ROS scavenging, anti-pruritic effects	Preclinical (in vivo)	[87,88,90,91]
Biomimetic EVs/vesicles	pab-miR-396a-5p, IL-17RA decoys, etomoxir	Native biocompatibility; biomimetic targeting; gene-regulatory cargo	Complex isolation; low yields; stringent quality control; safety characterization needed	pab-miR-396a-5p EVPs, NK-cell EV MNs, keratinocyte-derived apoptotic nanovesicles, Treg-exosome MNs; IMQ mouse model; reduced PASI-like scores and Treg modulation	Preclinical (in vivo)	[13,26,93,94]
Pure-drug n anocrystals	Tacrolimus, apremilast, tetrandrine	Carrier-free; maximal loading; improved solubility and retention	Ostwald ripening; requires high-energy wet milling	Tacrolimus nanocrystal MN, apremilast nanocrystal gel, tetrandrine nanocrystals; IMQ mouse model; CD4^+^FOXP3^+^ Treg expansion (tetrandrine)	Preclinical (in vivo)	[49,98,99,100]

**Table 3:** Conceptual comparison of current systemic biologics and microneedle-delivered tolerogenic nanovaccines for psoriasis.

Parameter	Systemic Biologics	MN-Delivered Tolerogenic Nanovaccines
Administration	Subcutaneous or intravenous injection [24,103]	Self-applied transdermal patch [36,38]
Mechanism	Receptor/cytokine blockade; intracellular pathway inhibition [2,16,133]	Antigen-specific tolerance induction or local immune reprogramming [17,19,44]
Efficacy (current evidence)	High and durable skin clearance (PASI75/90/100 in randomized trials) [2,10,24,138]	Preclinical only; no clinical efficacy data in psoriasis [3,45,46]
Durability	Requires continuous treatment; relapse after withdrawal [24,25,103]	Potential durable tolerance and relapse prevention [18,26,44]
Safety profile	Class-specific signals (infection, mucocutaneous candidiasis, inflammatory bowel disease); immunogenicity [2,10,138]	Local tolerability to be established; long-term safety unknown [40,45]
Development stage	Approved and in clinical use [2,10,24]	Preclinical; first-in-human studies not yet conducted [3,40,45]
Cost	High; reimbursement varies across regions [24,103]	Not assessable; depends on manufacturing scale and durability of response [45,47]

## Data Availability

The literature screening and filtering process is included in the Supplement File. Data sharing is not applicable to the review.

## Supplementary Materials

The following supporting information can be downloaded at: https://www.mdpi.com/article/10.3390/vaccines14090784/s1, Figure S1: Flowchart of the literature screening and filtering workflow derived from Pubmed.

## Abbreviations

AMP	antimicrobial peptide
APC	antigen-presenting cell
CD	cluster of differentiation
CD8^+^ TRM	tissue-resident memory T cell
CMC	carboxymethyl cellulose
CQA	critical quality attribute
CRISPR	clustered regularly interspaced short palindromic repeats
DC	dendritic cell
DEJ	dermal-epidermal junction
DFK	difelikefalin
DLQI	Dermatology Life Quality Index
DMN	dissolving microneedle
EV	extracellular vesicle
EVP	extracellular vesicle-like particle
FDA	Food and Drug Administration
FOXP3	forkhead box P3
GelMA	gelatin methacryloyl
GLP	Good Laboratory Practice
HA	hyaluronic acid
HAMA	methacrylated hyaluronic acid
HFMN	hydrogel-forming microneedle
IL	interleukin
IL-17RA	IL-17 receptor A
IMQ	imiquimod
JAK	Janus kinase
LNP	lipid nanoparticle
MMP	matrix metalloproteinase
MN	microneedle
MSN	mesoporous silica nanoparticle
MTX	methotrexate
NLC	nanostructured lipid carrier
NK	natural killer
PASI	Psoriasis Area and Severity Index
PCSK9	proprotein convertase subtilisin/kexin type 9
PDT	photodynamic therapy
PEGDA	poly(ethylene glycol) diacrylate
PGA	Physician Global Assessment
PMVE/MA	poly(methyl vinyl ether-co-maleic anhydride)
PRO	patient-reported outcome
PVA	polyvinyl alcohol
PVP	polyvinylpyrrolidone
QbD	quality by design
RORγt	RAR-related orphan receptor gamma t
ROS	reactive oxygen species
siRNA	small interfering RNA
SLN	solid lipid nanoparticle
STAT3	signal transducer and activator of transcription 3
TAC	tacrolimus
tDC	tolerogenic dendritic cell
Th17	T helper 17
TNF	tumor necrosis factor
Treg	regulatory T cell
TSDR	Treg-specific demethylated region
TSPBA	thioketal-containing phenylboronic acid-based crosslinker
TYK2	tyrosine kinase 2
