# Supplementary material for: Can Microneedle-Nanocarrier Platforms Deliver True Tolerogenic Immunotherapy for Psoriasis?
Source: Vaccines (Basel). 2026 Sep 8;14(9):784. doi: 10.3390/vaccines14090784 (PMC13611329; doi:10.3390/vaccines14090784)
Supplement: Supplementary file 1 [file vaccines-14-00784-s001.zip › vaccines-4528255-supplementary.pdf]

Search Strategy and Study Selection

The narrative review synthesizes peer-reviewed literature on microneedle-delivered nanocarriers for tolerogenic immunotherapy in psoriasis [1-3]. We searched PubMed/MEDLINE from inception to 1 August 2026 using Boolean queries built from two keyword blocks: (psoriasis) AND (microneedles OR nanoparticles OR liposomes OR nanocarriers OR drug delivery systems). The search strategy was:

("psoriasis"[MeSH Terms] OR "psoriasis"[Title/Abstract]) AND ("microneedles"[MeSH Terms] OR "microneedle"[Title/Abstract] OR "nanoparticles"[MeSH Terms] OR "nanoparticle"[Title/Abstract] OR "liposomes"[MeSH Terms] OR "liposome"[Title/Abstract] OR "nanocarriers"[Title/Abstract] OR "drug delivery systems"[MeSH Terms]).

Results were restricted to English-language original articles and reviews with available abstracts and full text; preprints were excluded. A total of 1066 records were identified.

A total of 1066 records were initially retrieved. After excluding 16 comments, 7 editorials, and 0 conference abstracts, 1043 records remained. After applying the "Abstract and Full Text" filter, 98 records were excluded, leaving 945 records. Four non-English records were then excluded, with no duplicates or preprints removed, resulting in 941 records for title/abstract screening. Two authors independently screened the titles and abstracts against predefined inclusion criteria, retaining 248 records for full-text assessment. After full-text evaluation, 70 records were excluded, and 178 references were finally included in the review. Disagreements were resolved by discussion and consensus, the sceening process is shown in Figure S1. Additional records were obtained by screening the reference lists of included articles; no journal-specific searches were performed.

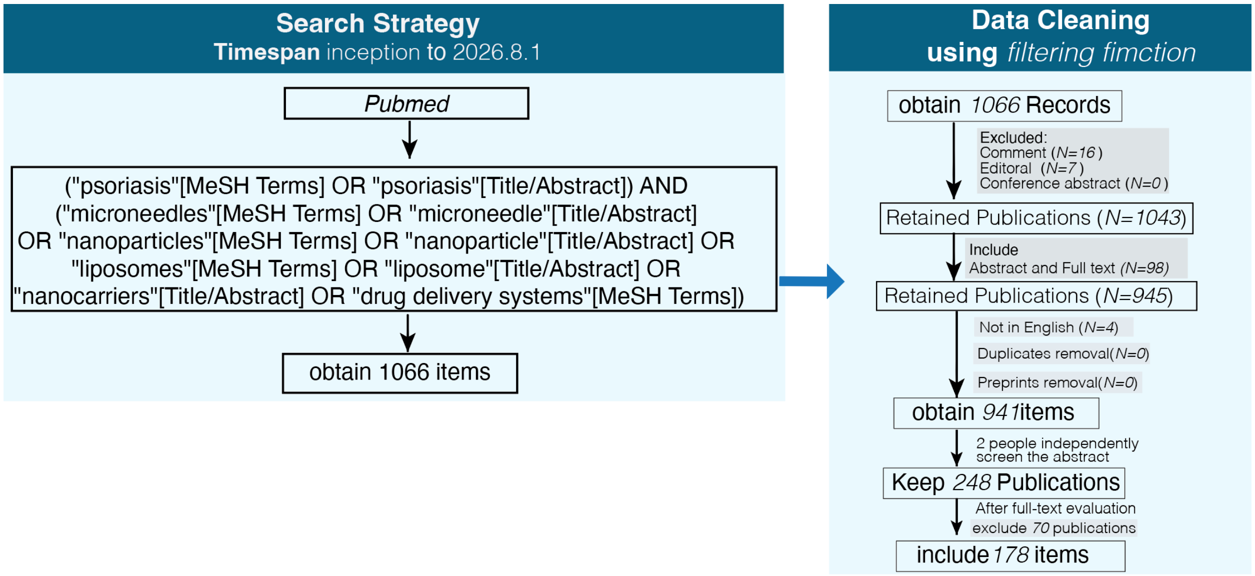

Figure S1 Flowchart of the literature screening and filtering workflow derived from Pubmed

References

1. Thakur, R.K.; Kumar, A.; Aggarwal, K.; Sood, N.; Khare, S.; Patel, P.; Das Kurmi, B. A complete sojourn on nanotechnological advancements and nanocarrier applications in psoriasis management. *Naunyn Schmiedebergs Arch Pharmacol* **2025**, *398*, 6453-6471, doi:10.1007/s00210-025-03804-w.
2. Silvestrini, A.V.P.; da Silva, U.J.; Morais, M.F.; Depieri, L.V.; Bentley, M. Integrating clinical challenges and nanotechnological based approaches for psoriasis therapy. *Biomater Adv* **2026**, *180*, 214625, doi:10.1016/j.bioadv.2025.214625.
3. Moawad, F.; Ruel, Y.; Pouliot, R.; Brambilla, D. Microneedle Technology in Psoriasis Management: Mechanistic Insights, Technological Innovation, Clinical Progress, and Challenges. *Adv Healthc Mater* **2026**, *15*, e04294, doi:10.1002/adhm.202504294.
